# Cells heal themselves: gene-edited hematopoietic stem cells restore frataxin deficiency in multiple organs

**DOI:** 10.1172/JCI211630

**Published:** 2026-10-15

**Authors:** Lucy R. Osborne

**Affiliations:** Departments of Medicine and Molecular Genetics, University of Toronto, Toronto, Ontario, Canada.

## Abstract

Friedreich’s ataxia (FRDA) is a triplet repeat expansion disorder with multisystem, progressive symptoms caused by reduced frataxin protein levels and compromised mitochondrial function. Restoration of frataxin protein is a focus of clinical trials involving adeno-associated viral vectors, but substantial barriers exist because of the immune response they elicit. In this issue of the *JCI*, Sivakumar et al. harnessed CRISPR/Cas9 editing of endogenous hematopoietic stem and progenitor cells (HSPCs) to excise the frataxin gene (*FXN*) repeat expansion ex vivo, followed by autologous transplantation. A single, systemic infusion of corrected HSPCs resulted in long-term engraftment and sustained multiorgan benefit in a humanized mouse model of FRDA. This improvement in brain, cardiac, and pancreatic symptoms, mediated by myeloid progeny of the engrafted stem cells, provides strong support for the potential effectiveness of this approach in humans and bypasses the need to correct the *FXN* deficiency in the affected tissue and cells directly.

## Friedreich’s ataxia etiology and symptoms

Friedreich’s ataxia (FRDA) is a rare, autosomal recessive disorder most commonly caused by a GAA triplet expansion in intron 1 of the frataxin gene (*FXN*), which leads to transcriptional silencing and FXN protein levels below 25% of those found in unaffected people (1). Effects on the body include neurodegeneration of the central and peripheral nervous system, resulting in progressive ataxia, dysarthria, and sensory neuropathy; hypertrophic cardiomyopathy frequently progressing to arrhythmias and congestive heart failure; and loss of insulin-secreting β cells in the pancreas, resulting in glucose intolerance or diabetes in the majority of patients (2). Symptoms usually become apparent in childhood or adolescence, with many individuals becoming reliant on wheelchair use, followed by death within three decades, most often due to cardiovascular pathologies.

FXN itself is a nuclear-encoded mitochondrial protein that is critical for iron homeostasis as well as ATP production through the electron transport chain (3). The FRDA-associated reduction in FXN disrupts the formation of iron-sulfur clusters, causing iron overload and oxidative stress sensitivity, particularly in mitochondria-rich organs such as the brain, heart, pancreas, and skeletal muscle (3). Mitochondria become fragmented, biogenesis is disrupted, antioxidants become depleted, and cells undergo an iron-dependent form of cell death called ferroptosis (4).

## Traditional approaches to therapeutic intervention

Although clinically identified more than 150 years ago (5) and molecularly defined three decades ago (1), there is no current effective therapy for those with FRDA. Most widely available treatments focus on improving quality of life, maximizing function, and reducing complications (2).

Recent efforts to develop new therapies have followed two avenues. The first targets modulation of downstream pathways such as iron homeostasis, oxidative stress, and mitochondrial biogenesis to ameliorate the effects of reduced FXN levels. Approved compounds in use or in active clinical trials include omaveloxolone, a nuclear factor erythroid 2–related factor 2 activator that reduces oxidative stress, and induces the broad activation of intracellular and mitochondrial antioxidative pathways (6); and high-dose resveratrol, a naturally occurring polyphenol that promotes mitochondrial biosynthesis (7). Numerous other compounds are in the development pipeline, such as iron chelators, epigenetic desilencers, ubiquitin-proteosome inhibitors, transcriptional inducers, and kinase inhibitors (8).

The second approach tackles the problem at its source, attempting to increase FXN levels through gene or protein replacement, molecular transcriptional stabilization or upregulation, and gene editing (8). Several gene replacement therapies are in clinical trials for cardiac tissues and the central nervous system, but they rely on delivery by adeno-associated virus (AAV) vectors. AAV vectors tend to display tissue-specific tropism but cannot be administered in multiple doses targeting different tissues due to the development of a robust adaptive immune response that can lead to suppression of transgene expression or severe adverse events (9). Delivery to the central nervous system also presents its own challenges, such as the vector’s penetration of the blood-brain barrier, dose-limiting toxicity, and a requirement for surgical or intrathecal administration.

An alternative to gene replacement is in vivo editing of the variant gene itself. This has been successfully demonstrated in other human disorders: hereditary angioedema and carbamoyl-phosphate synthetase deficiency have both been successfully treated in humans using a lipid nanoparticle delivery system rather than AAV (10, 11). Although this delivery route bypasses viral vector immunogenicity, lipid nanoparticles accumulate primarily in hepatocytes and have their own associated toxicity and reactivity issues (12).

## Harnessing hematopoietic stem cells for treatment

Another approach to genetic therapy has been the use of stem cell transplants derived either from a healthy donor or from the patients themselves and transferred to the patient after replacement of the disease gene ex vivo. A trial led by Stephanie Cherqui used lentiviral transduction of autologous CD34^+^ hematopoietic stem and progenitor cells (HSPCs) to replace the cystinosin gene, followed by transplantation back into the patient to successfully treat cystinosis, a lysosomal storage disorder causing multiorgan failure and premature death (13). The transfer of proteins from engraftment-derived macrophages or microglia-like cells to cystinosin-deficient cells was shown to occur through tunneling nanotubes — actin-based membranous connections between cells that allow cytosol-to-cytosol exchange (14).

In this issue, a study by Sivakumar et al. (15) from Cherqui’s group utilized this approach to provide a proof of principle for gene therapy in FRDA that was built upon a decade of foundational studies. In contrast to gene or protein replacement, their approach incorporated gene editing of HSPCs to remove the repeat expansion that suppresses *FXN* transcription to boost endogenous protein production. They had previously shown that transplantation of HSPCs from a WT mouse preserved brain, muscle, and cardiac function in a humanized mouse model of FRDA (YG8JR), which harbors a human *FXN* transgene with > 800 GAA repeats on a murine *Fxn*-null background (16, 17). The mechanism of rescue was similar to that demonstrated in the cystinosis therapy study, with transfer of functional FXN protein from engrafted HSPC-derived microglia or macrophage cells to FXN-deficient neurons, myocytes, and cardiomyocytes.

Another step laying the groundwork for the present study was optimizing CRISPR/Cas9 editing of the *FXN* GAA expansion in human cells to allow autologous transplant and avoid the need for immunosuppression that accompanies donor transplants. The group accomplished this first in lymphoblasts, then in CD34^+^ HSPCs isolated from peripheral blood (18), and finally in FRDA patient–derived induced pluripotent stem cells (iPSCs) in which neurons from gene-edited iPSCs showed rescue of apoptotic and mitochondrial phenotypes (19).

## Multiorgan improvement after engraftment of corrected cells

In the current study, Sivakumar et al. (15) take the autologous HSPC gene therapy approach to its conclusion, correcting endogenous HSPCs ex vivo using the CRISPR/Cas9 system to excise the GAA repeat from *FXN*, followed by autologous transplantation of the engineered cells back into irradiated YG8JR mice. This single, systemic infusion of gene-edited HSPCs resulted in long-term engraftment and sustained multiorgan benefit (Figure 1).

Cas9 nuclease was used in combination with two single mRNA guides to excise the *FXN* GAA repeat in Sca1^+^ HSPCs harvested from YG8JR mice. These cells are analogous to human CD34^+^ HSPCs. Ex vivo excision efficiency of the GAA repeat averaged 20.3% (range: 7.6%–30.5%). When transplanted into 2-month-old lethally irradiated YG8JR mice, cells derived from these edited, transplanted HSPCs showed widespread distribution across tissues, with chimerism ranging from 26.5% in spleen to 0.52% in the cerebellum and brain stem. When the GAA repeat excision efficiency was greater than 14%, engrafted mice showed increased human FXN tissue expression, reduced mitochondrial stress, and reduced inflammatory signaling.

Inflammation was mitigated in all tissues examined, with marked reduction of proinflammatory CD68^+^ macrophages in the pancreas and heart and absence of activated microglia in the cerebellum. Transcriptional profiling of cerebellar tissue demonstrated an enrichment of immune and inflammatory pathways in mice transplanted with edited HSPCs, confirming a targeted therapeutic effect on microglia-associated neuroinflammatory programs.

The transplantation of corrected HSPCs slowed or halted disease progression in the brain, heart, and pancreas, preserving tissue architecture at the point where engraftment took place, improving locomotor deficits, and modulating inflammatory environments that were contributing to cellular degeneration.

## The bright future of FRDA therapy

There is undoubtedly a lot of work to be done optimizing the editing efficiency of the repeat expansion, cell engraftment, and the timing of delivery, but the results reported by Sivakumar et al. are very encouraging and demonstrate that even with relatively limited editing and chimerism, there were concrete improvements in neurological, pancreatic, and cardiac function in the humanized mouse model of FRDA.

The timing of treatment delivery will be an important consideration when moving this approach into patients. In mice, treatment after pathological features of FRDA were already apparent improved gait and stabilized cerebellar architecture but did not reverse existing neuronal loss. Similarly, cardiac architecture was preserved but hypertrophy remained. Whether the therapeutic effects in the heart are enough to prevent congestive heart failure remains to be seen, since the YG8JR model develops a relatively mild cardiac phenotype (20). One of the surprising outcomes of the study was that pancreatic β cell numbers rebounded to WT numbers, demonstrating recovery of physiological function rather than stabilization.

The methodology of introducing the gene-editing components (Cas9 nuclease and two sgRNAs) via electroporation rather than using a viral vector or lipid nanoparticle has obvious benefits, but the risk of off-target editing remains a possibility, though a remote one given the high specificity of the guides. Advances in the development of RNA delivery, particularly the use of nonviral systems such as nanoparticles, exosomes, or hydrogels, in combination with physical techniques to penetrate the blood-brain barrier, offer promise for safer, more efficient gene therapy. Eventually, it may be optimal to deliver the gene-editing components directly to cells throughout the body, but in the meantime, targeting correction to HSPCs is an effective and practical alternative.

Finally, the correlation of gene-editing efficiency with the level of engraftment and therapeutic benefit demonstrated by Sivakumar et al. is particularly encouraging as it suggests that even modest increases in GAA excision efficiency could considerably improve chimerism and clinical efficacy upon transplantation.

## Conflict of interest

The author has declared that no conflict of interest exists.

## Figures and Tables

**Figure 1:**
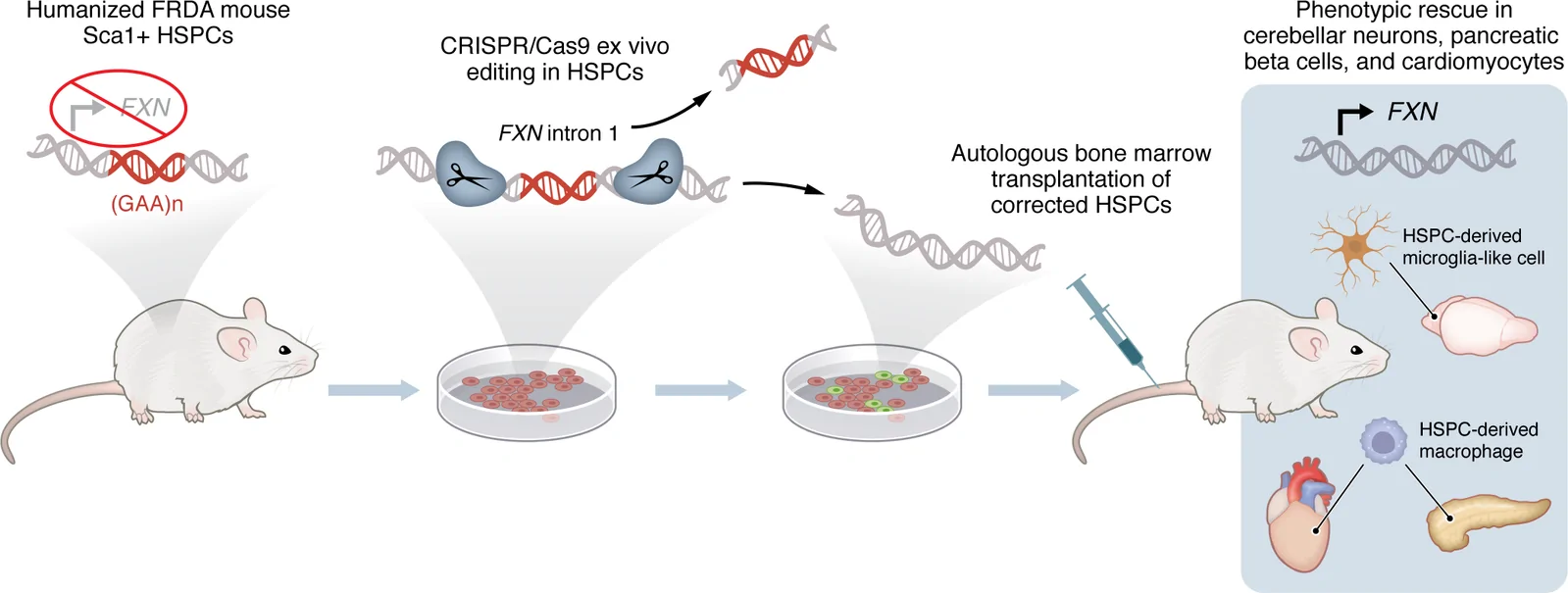
CRISPR/Cas9-edited HSPCs restore FXN deficiency in multiple organs in a humanized mouse model of FRDA. Sivakumar et al. (15) used ex vivo CRISPR/Cas9 editing to excise the FRDA GAA repeat expansion that suppresses *FXN* gene transcription in HSPCs harvested from a humanized FRDA mouse model. They performed a proof-of-principle study to demonstrate that a single systemic infusion of gene-edited HSPCs back into irradiated FRDA mice resulted in bone marrow engraftment and multisystemic benefits in cerebellum, heart, and pancreas. These effects, mediated by HSPC-derived macrophages and microglia-like cells, were driven by increased human FXN expression, reduced mitochondrial stress, and inflammatory signaling.

## Version 1 10/15/2026

Electronic publication
